# Lumbar puncture position in infants—a systematic review and meta-analysis

**DOI:** 10.1007/s00431-023-05137-3

**Published:** 2023-08-04

**Authors:** Ilari Kuitunen, Marjo Renko

**Affiliations:** 1 Institute of Clinical Medicine and Department of Pediatrics, University of Eastern Finland, Kuopio, Finland; 2 Department of Pediatrics, Kuopio University Hospital, Kuopio, Finland; 3 Department of Pediatrics, Mikkeli Central Hospital, Porrassalmenkatu 35-37, Mikkeli, 50100 Finland

**Keywords:** Lumbar puncture, Spinal tap, Meningitis

## Abstract

To analyze the optimal lumbar puncture position in infants. A systematic review and meta-analysis. Infants (age < 1 year). December 2022 in PubMed, Scopus, and Web of Science. Randomized controlled trials focusing on lumbar puncture positions were included. Other lumbar puncture position than standard lateral decubitus position. First puncture success and overall success rate. Secondary outcome was desaturation during puncture and procedure-related harms. Risk of bias 2.0 assessment was performed. Outcomes are reported as risk ratios (RR) with 95% confidence intervals (CI). We screened 225 abstracts, and six studies were included. Four studies compared sitting position, one study head elevated lateral position, and one study prone position to lateral position. Risk of bias was high in two studies. First puncture success rate in sitting position (RR 1.00, CI: 0.78–1.18; 2 studies) and overall success rate in sitting position were similar to lateral position (RR 0.97, CI: 0.87–1.17; 3 studies). First attempt success rate was higher in elevated lateral position (RR 1.48, CI: 1.14–1.92; 1 study) and in prone position (RR 1.09, CI: 1.00–1.17; 1 study).

*  Conclusion*: Sitting position seems to be equally effective in terms of first attempt and overall success in lumbar puncture than standard lateral position. Elevated lateral position and prone positions had better first attempt success than standard lateral position, but these were assessed only in one study each and thus further studies in these positions are needed.

*  Trial registration*: This review was registered in PROSPERO. ID: CRD42022382953.

**Table:** 

**What is Known:** *• Success rate in lumbar puncture has been poor and first attempt success rate has varied between 50 to 80% in literature.* *• Optimal lumbar puncture positions for infants have been debated between sitting and lateral decubitus position mostly.*	
**What is New:** *• This is the first meta-analysis focused on lumbar puncture positions in infants, and it found that sitting position was equal to standard lateral position.* *• Prone position and head elevated lateral positions had higher first puncture success rates, but these were assessed both only in one study, which creates uncertainty to the finding.*

**What is Known:**

*•  Success rate in lumbar puncture has been poor and first attempt success rate has varied between 50 to 80% in literature.*

*•  Optimal lumbar puncture positions for infants have been debated between sitting and lateral decubitus position mostly.*

**What is New:**

*•  This is the first meta-analysis focused on lumbar puncture positions in infants, and it found that sitting position was equal to standard lateral position.*

*• Prone position and head elevated lateral positions had higher first puncture success rates, but these were assessed both only in one study, which creates uncertainty to the finding.*

## Introduction

Lumbar puncture is among the most common invasive procedures in children. Especially neonates and infants have high need for lumbar punctures due to the highest rates of bacterial meningitis, as for example the infant bacterial meningitis rate in the UK has been reported to be 0.4 per 1000 births [1, 2]. The first attempt success rate of lumbar puncture is low and has varied between 50 and 80% in previous studies [3–5]. The rates of traumatic lumbar puncture (classified as red blood cell count > 10,000/µl) are higher in neonates than in infants [6].

Optimal lumbar puncture position in infants and neonates has remained controversial. Few observational studies assessing the success rate between different positions have found that sitting position might have a higher success rate [7–9]. However, there are also reports with lower success rates in sitting position [10]. Other alternative positions have rarely been studied in infants. Furthermore, the optimal puncture position has been evaluated by ultrasound in terms of how the puncture space opens in sitting position compared to lateral position. In these studies, sitting position has seemed to offer the widest opening angle for lumbar puncture [11, 12]. However, the clinical first attempt success rate of ultrasound-marked puncture sites in randomized controlled trials has been relatively comparable to standard palpation method [3, 13, 14]. A recent large randomized controlled trial (RCT) found that sitting position was associated with better success rate than standard lateral position [15]. Thus, we wanted to update the current evidence on optimal lumbar puncture position in infants.

The aim of this systematic review with meta-analysis was to compare first attempt and overall puncture success rates between different lumbar puncture positions in infants.

## Methods

### Search process

We searched PubMed, Scopus, and Web of Science databases on December 4, 2022. The following search phrase was utilized: “lumbar puncture” AND position AND (infant OR neonate OR newborn OR child OR pediatric). Reference lists of the included studies were also hand searched, and relevant articles included, if found. Search results were then uploaded to Covidence software (Covidence systematic review software, Veritas Health Innovation, Melbourne, Australia, 2022) for screening. Two authors independently screened the abstracts and the full texts. In cases of disagreement, a mutual consensus was searched by discussion.

### Inclusion and exclusion criteria

We included randomized controlled studies that compared any other lumbar puncture position to standard lateral decubitus position. We classified infants as children aged 0 to 364 days. We excluded all observational studies. We excluded studies with older children if data were not presented separately for infants. Furthermore, non-English studies and studies that did not present any original data were excluded.

### Outcome measures

Our main outcome measures were the rate of first attempt success and overall puncture success rate. In all analyses, alternative lumbar puncture positions were compared to standard lateral decubitus position. A lumbar puncture attempt is classified as needle perforating the skin. The definition for a successful puncture was that the fluid was clear and that red blood cell count was less than 10,000/µl. First attempt success rate is defined as obtaining successful cerebrospinal fluid sample with first puncture. Overall puncture success rate is defined as obtaining successful cerebrospinal fluid sample regardless of the number of attempts. Secondary outcomes were the rates of desaturation during the lumbar puncture and puncture-related adverse events.

### Data extraction

The following data were extracted by one author and verified by the other author from each included study to a predesigned Excel worksheet: authors, journal, country, setting, main outcome(s), secondary outcome(s), number of participants in each group, number of successful punctures, number or first attempt successful punctures, overall number of punctures, and adverse events.

### Risk of bias

Risk of bias was assessed according to Cochrane risk of bias 2.0 tool [16]. Risk of bias is presented for each individual study and as a summary plot per assessed domains. Figures were generated by robvis package [17].

### Statistics

This review has been conducted according to the guidelines in the Cochrane handbook of systematic reviews [18]. Studies were pooled together in meta-analysis. Random-effects model was chosen due to expected heterogeneity between the studies. Risk ratios with 95% confidence intervals (CI) were calculated with Mantel–Haenszel test. Publication bias is analyzed for all analyses where at least five studies are included [19]. Review Manager version 5.4.1 was used in all statistical analyses.

Evidence quality for all outcomes was assessed by the Grading of Recommendations, Assessment, Development and Evaluations [20]. This study has been reported according to the preferred reporting items in systematic reviews and meta-analyses 2020 (PRISMA) guideline and the checklist is found in the supplementary materials [21].

### Protocol registration

This protocol was registered to PROSPERO: ID CRD42022382953. It is available from https://www.crd.york.ac.uk/prospero/display_record.php?ID=CRD42022382953.

## Results

### Search

**Fig. 1:**
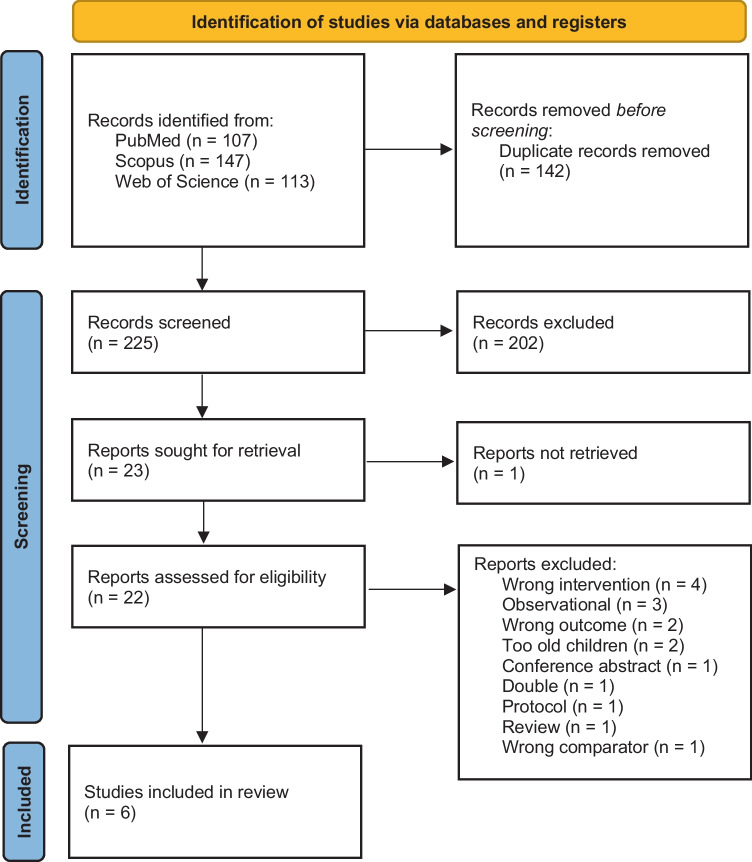
Flow chart of the study selection process

A total of 225 abstracts were screened. After further assessment of 22 full reports, 16 studies were excluded [22–37] and 6 studies included for systematic review and meta-analysis (Fig. 1) [15, 38–42]. All the six studies included were conducted in high-resource countries. Three studies were performed in neonatal intensive care units, two in operation rooms and one in an emergency department (Table 1). Interestingly, only one study reported the funding details and three studies had conflicts of interest statements (Table 1). In studies that reported the baseline characteristics, there were no substantial differences regarding age, weight, and gestational age of the infants (Table 2).

**Table 1:** Characteristics of the included studies

Study	Country	Setting	Intervention	Control	*N* participants	Primary outcome	Secondary outcome	Adverse events	COI	Funding
Apiliogullari et al. [40]	Turkey	Operation room before surgery	Elevated lateral position	Lateral position	106	Successful first attempt without redirection of the needle	N/A	Not specified	Not reported	Not reported
Guo et al. [39]	China	Neonatal intensive care unit	Prone position	Lateral position	171	Successful first attempt	Successful puncture	Puncture complications, changes in the vital signs during puncture	No COI	Not reported
Hanson et al. [30]	USA	Emergency department	Sitting position	Lateral position	167	Successful puncture (red blood cells less than 10,000/µl)	Successful first attempt (red blood cells less than 10,000/µl)	Procedure-related adverse events, such as desaturation, bradycardia, and respiratory distress	No COI	Not reported
Marshall et al. [32]	UK	Neonatal intensive care units	Sitting position	Lateral position	1076	Successful first attempt (red blood cells less than 10,000/µl)	CSF appearance and number of attempts	Safety metrics (cardiorespiratory stability and adverse event reporting)	No commercial related to this work	Reported
Vila et al. [41]	Spain	Operation room before surgery	Sitting position	Lateral position	30	Not specified	Not specified	Apnea or bradycardia	Not reported	Not reported
Weisman et al. [42]	USA	Neonatal intensive care unit	Sitting position	Lateral position	26	Hypoxemia during puncture	Puncture success	Hypoxemia, bradycardia	Not reported	Not reported

**Table 2:** Characteristics of the infants in the included studies

Study	Age			Weight			Gestational age	
	Intervention	Control	Intervention		Control	Intervention		Control
Apiliogullari et al. [40]	N/A	N/A	N/A		N/A	N/A		N/A
Guo et al. [39]	N/A	N/A	Mean 1460 g		Mean 1411 g	Mean 31.3 weeks		Mean 32.1 weeks
Hanson et al. [38]	Mean 37 days	Mean 41 days	N/A		N/A	N/A		N/A
Marshall et al. [15]	Median 1 day	Median 2 days	Median 3500 g		Median 3530 g	Median 40 weeks		Median 40 weeks
Vila et al. [41]	Mean 35 weeks	Mean 35 weeks	Mean 2300 g		Mean 2100 g	Mean 30.7 weeks		Mean 30.2 weeks
Weisman et al. [42]	Mean 4.9 h	Mean 5.2 h	Mean 2142 g		Mean 1973 g	Mean 33.5 weeks		Mean 33.7 weeks

### Risk of bias

**Fig. 2:**
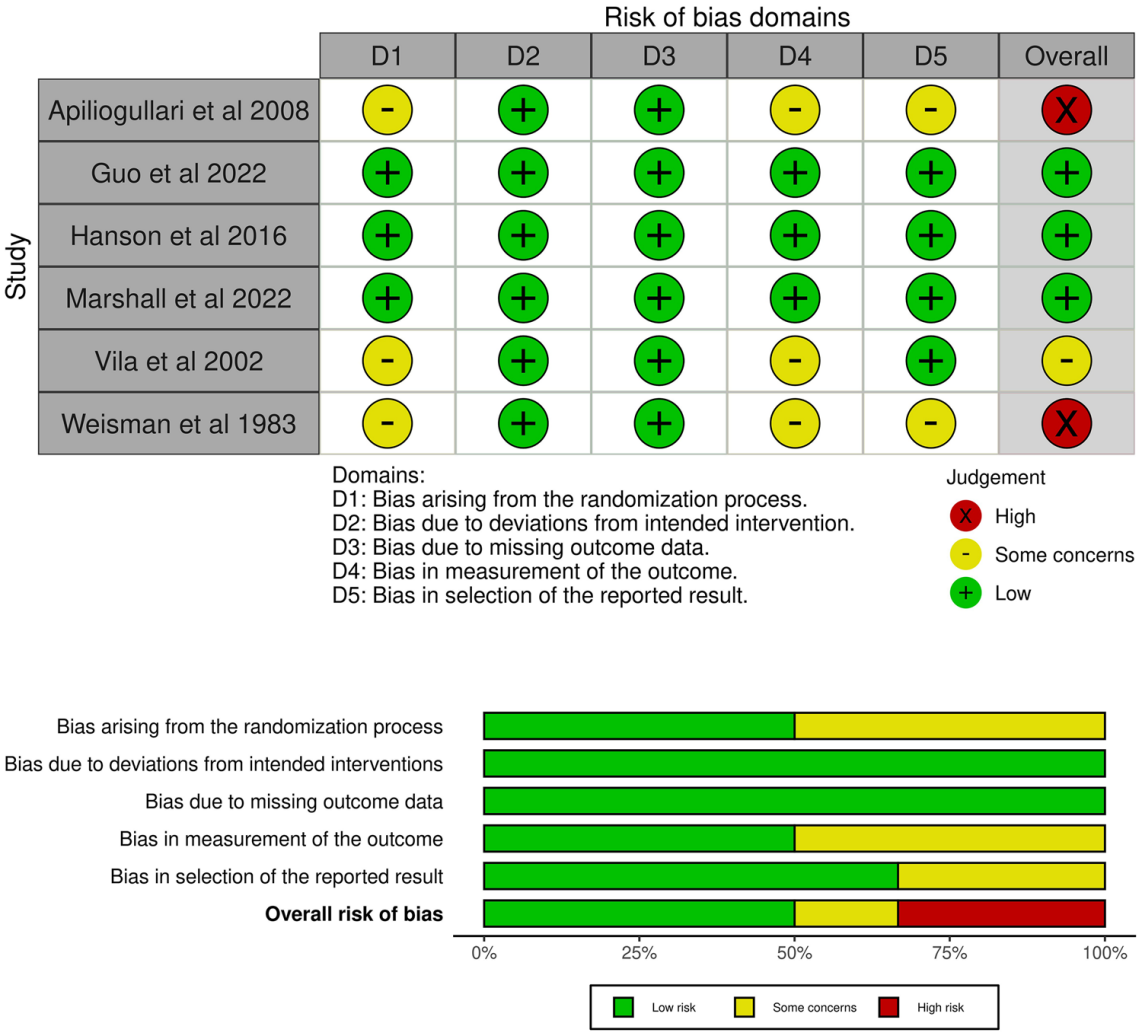
Risk of bias in the included studies assessed in five domains and overall

Risk of bias was assessed to be high in two studies; one had some concerns and three studies had low risk of bias (Fig. 2). Most biases arise from the randomization process and bias due to outcome measurement (Fig. 2, Table 3).

**Table 3:** Adverse events during lumbar punctures

	Desaturation	Puncture related	Other
Sitting position			
Hanson et al. [38]	One case of respiratory distress in lateral position group	No cases in either group	No reports of bleeding, apnea, or bradycardia in either group. No return visits to an ED within 1 month due to puncture complications
Marshall et al. [15]	Median lowest saturation 93% in sitting group (interquartile range 89 to 96), median lowest saturation 90% in lateral group (interquartile range 85 to 94), *p* < 0.001	One possible in sitting group, no cases in lateral group	Lowest mean heart rate in was 129.5 in sitting group and 127.0 in lateral group
Vila et al. [41]	Similar saturations in both groups	Not reported	No apneas, bradycardia, or hypotension episodes
Weisman et al. [42]	Lateral position group had longer durations of desaturation in pulse oximetry than sitting position	Not reported	Sitting group had lower intraesophagal pressure than lateral group
Prone position			
Guo et al. [39]	Not specified	Not specified	Prone group had 3 cases for adverse effect. Lateral position group had 24. The adverse effect for the prone group was less bleeding. Adverse effects of the standard position group were local hemorrhage, bradycardia, unstable transcutaneous oxygen saturation, etc.
Elevated lateral position			
Apiliogullari et al. [40]	Not specified	Not specified	No complications related to the spinal anesthesia were recorded

### First attempt success rate

**Fig. 3:**
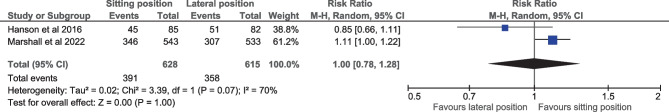
First attempt success rate in lumbar punctures. Sitting position compared to lateral position

Two studies [15, 38] with 1243 infants compared sitting position to lateral position (Fig. 3). The first attempt success rate was 62.3% in sitting position and 58.2% in lateral position (RR 1.00, CI 0.78–1.28, *I*^2^ 70%). Evidence quality was ranked as moderate (Table 4). One study [40] with 116 infants compared elevated lateral position (table tilted up 45 degrees from the head side) to normal lateral position and the first attempt success rate was higher in elevated lateral position (50/58, 86.2%) than in standard lateral position (28/48, 58.3%), RR 1.48 (CI 1.14–1.92). Evidence quality was ranked as low (Table 4). One study [39] compared prone position to standard lateral position with 171 infants in neonatal intensive care unit. Prone position had higher first attempt success rate (70/82, 85.4%) than standard lateral position group success rate (57/89, 64.0%), RR 1.33 (1.11–1.60). Evidence quality was ranked as low.

**Table 4:** Summary of findings table for main outcomes

Outcome	Relative effect (95% CI)	*N* participants (studies)	Evidence quality (GRADE)	Comments
First puncture success				
Sitting position	1.00 (0.78–1.28)	1,243 [2]	Moderate*	Two largest studies and least issues with risk of bias. Imprecision in the results
Elevated lateral position	1.48 (1.14–1.92)	106 [1]	Low**	Only one study assessed this
Prone position	1.33 (1.11–1.60)	171 [1]	Low**	Only one study assessed this
Overall success				
Sitting position	0.97 (0.87–1.09)	223 [3]	Low**	Three small trials, high risk of bias
Prone position	1.09 (1.00–1.17)	171 [1]	Low**	Only one study assessed this
Desaturation	Not pooled		Very low***	Very limited reporting
Puncture-related adverse events	Not pooled		Very low***	Very limited reporting

*Downgraded due to imprecision

**Downgraded due to risk of bias and uncertainty in the estimates as only one study assessed this intervention

***Downgraded due to risk of bias, clear heterogeneity in the outcome definitions, and lack of adverse event reporting

### Overall success rate

**Fig. 4:**
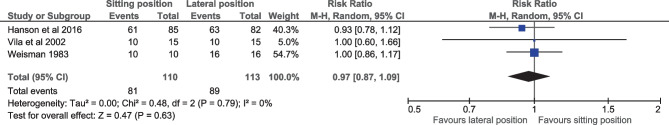
Overall success rate in lumbar punctures. Sitting position compared to lateral position

Three studies [38, 41, 42] with 223 infants compared the overall success rate between sitting and lateral position (Fig. 4). The overall success rate was 73.6% in sitting position and 78.8% in lateral position (RR 0.97, CI 0.87–1.09, *I*^2^ 0%). Evidence quality was ranked as low. One study [39] with 171 infants compared prone position to lateral position. Overall success rate was 97.6% (80/82) in prone position and 89.9% (80/89) in lateral position, RR 1.09 (CI 1.00–1.17). Evidence quality was ranked as low.

### Adverse events

Adverse events were reported heterogeneously between the studies (Table 3). One study reported that median lowest saturations were lower in the standard lateral position than in the sitting position [15] and another study reported longer durations of desaturation in lateral position [42]. Another study reported similar saturations in both groups [41]. Puncture-related adverse events were rare in all positions, as only one study reported a single case of puncture site hemorrhage (Table 3).

## Discussion

We found in this systematic review low- and moderate-quality evidence that sitting position is equally effective as standard lateral position in terms of overall success and first attempt success in lumbar punctures performed to infants aged less than 12 months. In addition, we found low-quality evidence that elevated lateral position and prone position had higher first attempt success rates than standard lateral position, but these positions were analyzed only by one study each. All positions seemed to have low rates of adverse events and desaturation during the punctures.

We did not identify any previous meta-analysis on the positions during lumbar punctures in infants. Our search retrieved three non-randomized studies that were excluded from this meta-analysis. All of these three assessed sitting position in comparison to lateral position and found that the success rate in sitting position was higher than in the standard lateral position [7–9]. The systematic review by Hart et al. stated that the different positions seemed to be equally effective and better quality evidence is needed. However, the authors did not pool the results or conduct any critical appraisal [43].

The success rates in the included studies varied between 58.2 and 100%. This causes clear heterogeneity in the results. Three studies were conducted in neonatal intensive care units, and these had both the lowest and highest success rates [15, 39, 42]. The original studies did not control for the experience of the lumbar puncture performer in their analyses. Furthermore, operation room lumbar punctures were conducted by anesthesiologists, who typically use routinely lumbar punctures in their daily job while conducting neuraxial anesthesia [40, 41]. In the three studies that were carried out in the neonatal units, the puncture was performed by a pediatrician. One of the studies was conducted in the emergency department, where the setting is completely different from an operating room or neonatal intensive care unit, and the punctures were performed by pediatricians, family medicine doctors, or emergency medicine doctors [38].

One possible confounder to the results could have been the type of analgesia or procedural sedation used. Two of the included studies did not comment on the pain relief or sedation used [38, 42]. Two studies used non-nutritive sucking, topical analgesic cream/gel, and sucrose gel as analgesic [15, 39]. One study used topical analgesic cream/gel and midazolam [40], and one study used topical analgesia with nitric oxide inhalation [41]. Thus, all the included studies were conducted on awake patients, with typical non-medical and topical analgesic methods; this does not cause any notable issues to the pooling and synthesis of these studies.

Our main strength is that we did not have protocol deviations. Furthermore, we were able to perform a systematic synthesis of an important topic. Most limitations arise from the limited reporting of the original studies included in this review. Only three of the included studies were judged to have low risk of bias; thus, the reporting quality was limited. Due to the low number of included studies, we did not conduct sensitivity or publication bias analysis. Marshall et al. defined the success rate as first procedure and did not clearly specify the number of attempts, whereas Hanson et al. defined the first puncture success as first attempt success rate. Heterogeneity was a notable limiting factor as for example the study settings were differing (neonatal intensive care unit vs emergency department), and this likely increased the heterogeneity and decreases the validity of the results. Furthermore, the included studies had heterogenous reporting especially in adverse events. Therefore, we decided not to pool these together in meta-analysis. Due to these factors, the evidence quality remained either very low or low mostly.

Continuous research effort is needed to improve the success rates in lumbar punctures in infants. Further studies are needed to confirm the findings of all modified positions in relation to standard lateral positions before clear recommendations on the optimal position can be given. Ultrasound was believed to be a promising tool for help in lumbar punctures, but the success rates in randomized controlled trials have not been superior to standard methods in infants [44]. There are some reports on the use of bioimpedance needles that are able to detect the cerebrospinal fluid and could thus guide more precisely the optimal depth of the puncture. However, bioimpedance needles have yet not been assessed in randomized trials nor in infants [45].

## Conclusion

We found low- to moderate-quality evidence that sitting position seems to be equally effective in terms of first attempt and overall success in lumbar puncture than standard lateral position. Elevated lateral position and prone positions had better first attempt success than standard lateral position, but these were assessed only in one study each and the evidence quality was low. Further studies on optimal lumbar puncture position are needed before concluding which should be the preferred position.

## Data Availability

All data generated during review process available upon request.
